# Catechol-O-Methyltransferase val158met Polymorphism Predicts Placebo Effect in Irritable Bowel Syndrome

**DOI:** 10.1371/journal.pone.0048135

**Published:** 2012-10-23

**Authors:** Kathryn T. Hall, Anthony J. Lembo, Irving Kirsch, Dimitrios C. Ziogas, Jeffrey Douaiher, Karin B. Jensen, Lisa A. Conboy, John M. Kelley, Efi Kokkotou, Ted J. Kaptchuk

**Affiliations:** 1 Division of General Medicine and Primary Care, Beth Israel Deaconess Medical Center and Harvard Medical School, Boston, Massachusetts, United States of America; 2 Program in Placebo Studies, Beth Israel Deaconess Medical Center and Harvard Medical School, Boston, Massachusetts, United States of America; 3 Department of Gastroenterology, Beth Israel Deaconess Medical Center and Harvard Medical School, Boston, Massachusetts, United States of America; 4 School of Psychology, University of Plymouth, Devon, United Kingdom; 5 Department of Internal Medicine, University of Athens, Athens, Greece; 6 Department of Surgery, Johns Hopkins, Baltimore, Maryland, United States of America; 7 Department of Psychiatry, Massachusetts General Hospital, Harvard Medical School, Boston, Massachusetts, United States of America; 8 Endicott College, Beverly, Massachusetts, United States of America; The Chinese University of Hong Kong, Hong Kong

## Abstract

Identifying patients who are potential placebo responders has major implications for clinical practice and trial design. Catechol-O-methyltransferase (COMT), an important enzyme in dopamine catabolism plays a key role in processes associated with the placebo effect such as reward, pain, memory and learning. We hypothesized that the COMT functional val158met polymorphism, was a predictor of placebo effects and tested our hypothesis in a subset of 104 patients from a previously reported randomized controlled trial in irritable bowel syndrome (IBS). The three treatment arms from this study were: no-treatment (“waitlist”), placebo treatment alone (“limited”) and, placebo treatment “augmented” with a supportive patient-health care provider interaction. The primary outcome measure was change from baseline in IBS-Symptom Severity Scale (IBS-SSS) after three weeks of treatment. In a regression model, the number of methionine alleles in COMT val158met was linearly related to placebo response as measured by changes in IBS-SSS (p = .035). The strongest placebo response occurred in met/met homozygotes treated in the augmented placebo arm. A smaller met/met associated effect was observed with limited placebo treatment and there was no effect in the waitlist control. These data support our hypothesis that the COMT val158met polymorphism is a potential biomarker of placebo response.

## Introduction

Placebo treatments have been shown to lead to clinical improvement in a broad spectrum of disorders [1]. Although advances have been made in understanding the neurobiology of placebo [2], our understanding of the genetic modulators of the placebo response remains a critical knowledge gap. Identifying the characteristics of placebo responders and non-responders is key to managing underlying placebogenic factors for patient benefit and to optimizing the design and interpretation of clinical trials.

Studies investigating brain activity associated with placebo response in pain, Parkinson's disease and depression, point to dopamine as a possible integrator of the placebo response [2], [3], [4], [5]. Dopamine is a catecholamine synthesized from tyrosine by tyrosine hydroxylase and dopamine decarboxylase. Once synthesized, dopamine is packaged into presynaptic vesicles and released into the synaptic cleft upon depolarization. Dopamine is cleared from the synapse either by the dopamine reuptake transporter (DAT), or degradation by monoamine oxidases A and B, or catechol-O-methyltransferase (COMT). Whereas reuptake is the primary mechanism of dopamine clearance in the striatum, in the prefrontal cortex, DAT is less abundant, rendering COMT activity critical in regulating prefrontal dopamine signaling [6], [7].

Among the genetic polymorphisms in the dopamine pathway, the COMT val158met polymorphism has been studied most extensively in clinical trials for its potential association with treatment responses. COMT val158met, is a G to A transition leading to amino-acid substitution at codon 158 in the transmembrane form of the enzyme [8]. The methionine isoform has reduced thermostability, resulting in a three to four-fold decrease in activity relative to the ancestral valine isoform [9]. This functional polymorphism has been correlated with variations in memory function [10], [11], cognition [12], attentional processing [13], affect [14], confirmation bias [15], pain processing and sensitivity [16], [17], [18], [19]. Met/met individuals have higher levels of performance in cognitive tests, which measure executive function as well as increased sensitivity to experimental and chronic pain relative to val/met and val/val individuals [16], [19], [20], [21], [22].

Given that COMT has an effect on dopamine levels in the prefrontal cortex, a brain region activated during placebo response, we hypothesized that the functional COMT val158met polymorphism is a placebo response variant. To our knowledge, no previous study has reported an association between COMT and response to placebo treatment in irritable bowel syndrome (IBS). IBS, a common gastrointestinal disorder affecting 10 to 15% of North Americans [23], [24] is characterized by abdominal pain or discomfort associated with altered bowel function, bloating, and a sensation of incomplete evacuation after bowel movements [25], [26]. IBS is a condition known to have a high placebo response rate and meta-analyses report an average placebo induced global improvement of approximately 40% [27], [28], [29].

Previously, our team investigated IBS placebo responses in a clinical trial which had three arms: 1) a no-treatment arm that controlled for regression to the mean and normal fluctuations in illness (“waitlist”), 2) a placebo treatment arm which used a validated placebo acupuncture device administered in a business-like no frills clinical context (“limited placebo”), and 3) a limited placebo arm augmented with a supportive warm provider who expressed confidence in the effectiveness of the treatment (“augmented placebo”). Overall, we found a robust clinical response to the placebo treatments; validated IBS outcome measures showed augmented placebo was significantly more effective than limited placebo, which in turn was more effective than the no-treatment waitlist control [30].

Our parent IBS clinical trial was ideal for the study of genetic associations with placebo effects because it included a waitlist (observation alone) control and two different “doses” of placebo (limited and augmented). The waitlist control allowed for separation of regression to the mean and the natural waxing and waning of illness from the effects of placebo treatment. The two types of placebo interventions, limited and augmented, created a comparison of incremental components of the placebo effect in a manner that could be considered analogous to dose dependent.

Here we present the first study to support the hypothesis that the COMT val158met polymorphism is a potential genetic marker of placebo response.

## Materials and Methods

### Study populations

A randomized clinical trial investigating placebo effects in IBS patients (Trial registration – NCT00065403) was conducted. Details of the design and outcomes of the trial are provided elsewhere [30], [31]. The 3-week trial enrolled 262 patients (75% women) ≥18 years and diagnosed by IBS Rome II criteria score of >150 on the Irritable Bowel Syndrome Symptom Severity Scale (IBS-SSS) [32].

### Randomization and Interventions

Patients were randomized to one of three treatment arms: (1) no-treatment control (“waitlist); (2) placebo acupuncture (“limited”); (3) placebo acupuncture plus a supportive patient-provider (“augmented”). A validated sham acupuncture device was used to deliver placebo acupuncture in 20 minute sessions, twice weekly for three weeks.

### Ethics Statement

A subgroup of patients (n = 112) gave consent for genetic analysis from blood samples included in this study. The Institutional Review Board at Beth Israel Deaconess Medical Center (Boston, MA) approved the main study and the genetic follow-up study presented here. All studies were conducted in accordance with the Declaration of Helsinki. Participants provided written consent for this genetic study. The ethics committee approved this procedure.

### Outcomes

Three validated IBS research measures from the previous trial, which assessed clinical outcomes, were used in this study. The primary outcome measure was the IBS-SSS, which consists of five 100-point scales (abdominal pain severity, abdominal pain frequency, abdominal distention severity, dissatisfaction with bowel habits, and quality of life disruption) that contribute equally to the final score, yielding a theoretical range of 0–500 [32]. Higher scores reflected a more severe condition. IBS-SSS was measured at baseline and after 3 weeks of treatment. Change in IBS-SSS was determined by subtracting 3-week IBS-SSS score from baseline IBS-SSS. We selected IBS-SSS as our primary outcome because the two secondary measures, Adequate Relief (AR) and the Global Improvement Scale (GIS) did not have baseline measures. Adequate Relief was assessed by a single dichotomous question at 3-weeks, which asked: “Over the past week have you had adequate relief of your IBS symptoms?” [33]. The GIS asked: “Compared to the way you felt before you entered the study, have your IBS symptoms over the past 7 days been: (1) = substantially worse, (2) = moderately worse, (3) = slightly worse, (4) = no change, (5) = slightly improved, (6) = moderately improved, or (7) = substantially improved” [34], [35]. These latter two measures were considered secondary because they do not have baseline assessments, thus opening the door to regression artifacts [36]. To mitigate this problem, we controlled for IBS-SSS baseline scores in analyses of AR and GIS.

### Genotyping

Of the 262 original study participants, 112 gave consent for genetic screening. Eight patients missing IBS-SSS data at 3-weeks were excluded from the analyses. Two additional patients were missing data for AR and GIS and were excluded from analysis of AR and GIS. Genomic DNA was extracted from whole blood using Qiagen Blood kit (Valencia, CA) following the manufacturer's protocol. Based on the association of COMT SNP rs4633 with COMT expression and activity [37], this SNP was genotyped in addition to rs4680 (val158met). TaqMan SNP Genotyping assays for rs4680 (val158met) and rs4633 were purchased from Applied Biosystems, (Foster City, CA). Quantitative PCR was performed at the Biopolymers Facility at Harvard Medical School, (Boston, MA) following the manufacturer's protocol on an Applied Biosystems 7900HT instrument, using SDS version 2.4 software.

### Statistical Analysis

Hardy–Weinberg Equilibrium (HWE) and Linkage Disequilibrium were calculated using the Online Encyclopedia for Genetic Epidemiology studies [38], [39]. Statistical analyses were performed using IBM SPSS Statistics version 20 (Chicago, IL).

We used an additive genetic model to investigate the linear effect of increases in the presence of the COMT met allele. We created a variable, “COMT genotype”, that coded each patient's val158met genotype as follows: 1 = met/met; 0 = val/met; −1 = val/val. Using standard coding for polynomial trends, we used multiple regression to examine linear and quadratic effects of COMT genotype (number of met alleles) on placebo responses as measured by changes from baseline IBS-SSS, and on AR and GIS. We controlled for initial disease severity by including baseline IBS-SSS as a covariate in the regression models. In addition, we created variables to test for linear and quadratic effects of treatment, conceiving these as ascending “doses” of non-specific effects (waitlist, limited placebo, augmented placebo) to test for interactions between COMT genotype effects and treatment received.

## Results

The clinical and demographic characteristics of the subset of genotyped patients (n = 104) relative to the original clinical trial (n = 262) [30] are shown in Table 1. Age, gender, race and marital status of the genotyped patients did not differ significantly from the distribution in the original study; duration, IBS type and baseline IBS-SSS were also similar. Eighty percent of the genotyped patients were women and 94% were white. Furthermore there were no significant differences in demographics and disease characteristics of genotyped patients across the COMT val158met genotypes. The number of patients genotyped and analyzed from each treatment arm (waitlist, 29%; limited, 32%; and augmented, 39%) was similar to the overall distribution in the original trial (waitlist, 33%; limited, 34%; and augmented, 33%).

**Table 1:** Baseline characteristics of genotyped IBS patients compared to participants in the parent randomized clinical trial.

Characteristics	All IBS patients* (n = 262)	Genotyped patients (n = 112)	p value	met/met (n = 20)	val/met (n = 55)	val/val (n = 29)	p value
Demographics							
Age in years (SD)	38 (14)	37 (13)	0.53	40 (12)	36 (13)	38 (14)	0.44
Women (%)	218 (83)	83 (80)	0.51	16 (80)	45 (81)	22 (76)	0.82
White (%)	252 (96)	98 (94)	0.17	20(100)	52 (95)	26 (89)	0.20
Married/living together (%)	101 (39)	32 (31)	0.42	8 (42)	16 (41)	8 (33)	0.79
**IBS symptoms and baseline characteristics**							
Type of IBS			0.56				0.80
Constipation (%)	74 (28)	35 (34)		5 (25)	21 (39)	9 (31)	
Diarrhea (%)	65 (25)	18 (17)		4 (20)	8 (15)	6 (21)	
Mixed (%)	139 (53)	40 (38)		11 (55)	25 (46)	14 (48)	
Disease for >5 years (%)	103 (39)	63 (61)	0.18	12 (60)	34 (62)	17 (59)	0.96
Baseline IBS-SSS (SD)	273 (73)	270 (66)	0.72	291 (78)	261 (64)	272 (61)	0.23

* Participants in parent clinical trial previously reported [30].

Based on its association with rs4680, COMT expression and enzymatic activity we also genotyped rs4633 [37]. Rs4633 was found to be in strong linkage disequilibrium with rs4680 (D'  = .94 and r2 = .9), such that the two SNPs were almost perfectly correlated: met/met, val/met and val/val of rs4680 corresponded to the T/T, T/C and C/C of rs4633. Therefore we focused on COMT val158met in further analyses. The minor allele frequency of COMT val158met was 0.46 and the SNP was in Hardy–Weinberg Equilibrium (p = .502).

In this study, IBS Symptom Severity Scale (IBS-SSS) was our *a priori* primary clinical outcome. IBS-SSS is a multidimensional measure that captures the full spectrum of IBS disease including abdominal pain severity, abdominal pain frequency, abdominal distention severity, dissatisfaction with bowel habits, and disruption in quality of life and has a theoretical range of 0–500 [32].

Both linear and quadratic effects of COMT alleles and treatment arm (waitlist, limited, augmented) were tested. As there were no significant main effects or interactions involving quadratic tests, results reported here are for a trimmed model with only linear effects and interactions included.

### IBS-Symptom Severity Scale (IBS-SSS)

**Figure 1:**
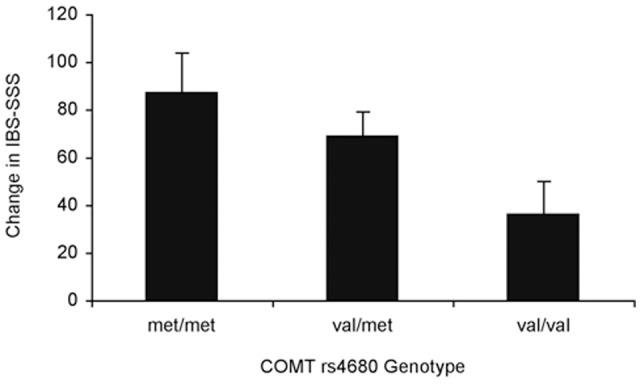
Effect of COMT genotype on change in IBS-SSS. Number of val158met met alleles showed a significant linear effect on IBS-SSS (beta = 0.17; p = .032). IBS-SSS includes abdominal pain severity, abdominal pain frequency, abdominal distention severity, dissatisfaction with bowel habits, and disruption of quality of life. Change in IBS-SSS  =  (IBS-SSS at baseline – IBS-SSS at 3-weeks). Regression model included COMT genotype (number of met alleles) and baseline IBS-SSS. Error bars indicate the standard error of the mean. N = 104.

Change in IBS-SSS was associated with a main effect of COMT genotype (number of met alleles) (Figure 1). Number of COMT val158met met alleles showed a significant linear effect on IBS-SSS (beta = 0.17; p = .032). Patients with the met/met genotype had the greatest level of improvement whereas val/val patients had the least, and val/met patients were intermediate (Figure 1). As in the original study [30], there was a main effect of treatment arm on improvement, with patients in the augmented treatment improving most and those on waitlist improving least (beta = 0.19, p = .019).

**Figure 2:**
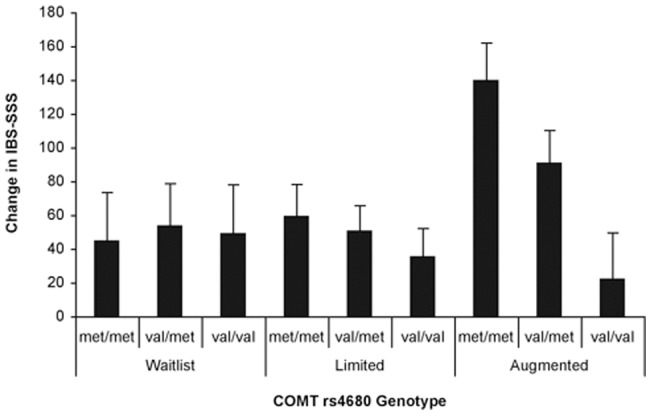
Interaction effect of COMT genotype and treatment arm on change in IBS-SSS. The interaction between COMT genotype (number of met alleles) and treatment arm was statistically significant (beta = 0.17; p = .035). Regression model included the following parameters: COMT genotype (number of met alleles), treatment arm, baseline IBS-SSS and their interaction (COMT genotype x treatment arm). Error bars indicate the standard error of the mean. N = 104.

These linear effects on IBS-SSS were qualified by significant interactions between COMT genotype and treatment arm (beta = 0.17; p = .035). COMT predicted responses in only the augmented placebo arm. In response to a high dose placebo treatment, patients homozygous for met alleles (met/met), showed the greatest improvement; val homozygotes (val/val) showed the least improvement and heterozygote (val/met) patients had an intermediate response (Figure 2).

### Adequate Relief

**Figure 3:**
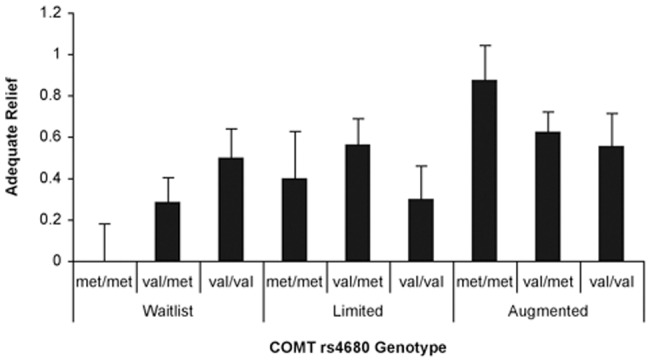
Interaction effect of COMT genotype and treatment arm on Adequate Relief. The interaction (COMT genotype x treatment arm) was statistically significant (beta = 0.25; p = .009), but not COMT genotype (beta = 0.01; p = .954). Adequate Relief was assessed by a single dichotomous categorization at three weeks, which asked patients: “Over the past week have you had adequate relief of your IBS symptoms?” Regression model parameters included: COMT genotype (number of met alleles), treatment arm and their interaction (COMT genotype x treatment arm). Error bars are standard error of the mean. N = 102.

The secondary validated measure of Adequate Relief uses a single dichotomous question at 3-weeks which asked: “Over the past week have you had adequate relief of your IBS symptoms?” [33]. The responses were coded as 0 = No, I did not have Adequate Relief and 1 = Yes, I had Adequate Relief. As expected from the previous study, there was a significant linear effect for treatment arm (beta  = 0.32, p = .001), in which patients in the augmented placebo arm showed most improvement and those in the waitlist arm showed the least [30]. Although there was no main effect for COMT genotype, there was a significant interaction between COMT genotype and treatment arm (beta  = 0.25, p = .009). In the augmented placebo arm, the met/met patients reported an average Adequate Relief score of 0.88 compared to 0.63 for val/met patients and 0.56 for val/val patients (Figure 3). Conversely, in the waitlist group, val/val patients had an average score of 0.50, whereas the met/met patients had an average score of 0.00, that is no met/met patients on the waitlist arm reported Adequate Relief.

### Global Improvement Scale

The other secondary validated measure was Global Improvement Scale (GIS) which asked: “Compared to the way you felt before you entered the study, have your IBS symptoms over the past 7 days been: (1) = substantially worse, (2) = moderately worse, (3) = slightly worse, (4) = no change, (5) = slightly improved, (6) = moderately improved, or (7) = substantially improved” [34]. As in the case of IBS-SSS and Adequate Relief, there was an expected significant linear effect for treatment arm (beta  = 0.31, p = .002), in which patients in the augmented placebo arm showed most improvement and those in the waitlist arm showed the least [30]. There was a trend in improvement associated with COMT genotype (beta = 0.18, p = .063) but no interaction of COMT genotype with treatment arm (beta = 0.07, p = .477).

## Discussion

In this study, we demonstrated that IBS patients homozygous for the COMT val158met methionine allele (met/met) were the most responsive to placebo treatment. Heterozygous (val/met) patients showed an intermediate response, and homozygous valine (val/val) patients showed essentially no placebo mediated symptom improvement. To our knowledge, this is the first study to demonstrate genetic modulation of true placebo effects disassociated from changes related to disease natural history and regression to the mean. It is also the first study to demonstrate a relationship between different levels of placebo treatment and COMT genotype.

Our regression analysis showed that as the number of COMT val158met met alleles increased progressively from 0 to 1 to 2, and COMT activity decreased, theoretically making more dopamine available in the prefrontal cortex, placebo responses increased in a linear fashion. Further, there was a significant interaction between number of methionine alleles and placebo treatment arm. Examination of the interactions illustrated in Figures 2 and 3, showed that the relationship between number of COMT met alleles and placebo response occurred primarily in the augmented placebo arm, with a smaller effect in the limited placebo arm, and no effect (IBS-SSS) or a reverse effect (Adequate Relief) in the waitlist control arm. Taken together, these results strongly suggest that COMT val158met, specifically the met/met genotype, is a potential marker for placebo responders in IBS. Furthermore the finding that this genotype is associated with a positive outcome only in groups given a placebo (and not in the waitlist control group) is of particular importance, as it indicates that it is a predictor of the placebo effect, not just improvement in general.

To date, the search for placebo biomarkers has been elusive. The three previous studies we are aware of which looked for a genetic link to the placebo response lacked the critical no-treatment control [21], [40], [41]. Neither of the two studies that examined the relationship between COMT and placebo response found a statistically significant relationship [21], [41]. The third study on severe anxiety disorder (SAD) found that polymorphisms in two serotonin-related genes, the serotonin transporter-linked polymorphic region (5-HTTLPR) and the G703T polymorphism of tryptophan hydroxylase-2, had a significant effect on placebo [40]. However the involvement of the amygdala and these two serotonin-related enzymes suggests that these associations may be unique to SAD and not necessarily generalizable to the placebo response mechanism. Furthermore in the absence of a no-treatment control that would have allowed a clear separation between placebo effects and such phenomenon as regression to the mean, spontaneous remission or the natural waxing and waning of illness, these studies could not rule out whether their findings were a result of a genuine psychobiological process or merely improvement that would have happened without placebo treatment.

Four other placebo-controlled studies designed to examine the effects of tolcapone, a COMT inhibitor, looked at the association between COMT val158met genotype, cognition and information processing [42], [43], [44], [45]. Mentioned *en passant* in their reports was the observation that the met/met subjects had a statistically significant placebo effect, whereas the val/val subjects were unresponsive to placebo treatment. Although this research paradigm is distinctly different from IBS, these findings support our hypothesis and suggest that the COMT mediated placebo response pathway might be generalizable across multiple conditions. Nonetheless, other replications of our study that prospectively hypothesize a COMT involvement with placebo effects and include a no treatment control will be critical to confirm our findings.

Positioned at the intersection of catecholamine metabolism, memory [46], reward processing [47], confirmation bias [15] and pain regulation [16], COMT presents an interesting candidate for a placebo response marker. The inverted U-shaped dopamine response curve, reported in many COMT functional studies suggests a narrow range for optimum dopamine cortical function [48], [49]. One might therefore hypothesize that drugs that interfere with the dopaminergic pathway might affect the observed response to treatment by shifting the component of the treatment response resultant from placebo. Indeed several studies that examine the effect of amphetamine [41] or tolcapone [42], [43], [44], [45] relative to a placebo treatment have observed that met/met subjects tend be unresponsive or worsen relative to placebo response when treated with these drugs, suggesting there is an optimum level of dopamine associated with placebo effect. Identifying biological characteristics of placebo responders and non-responders could be key to managing underlying placebogenic factors to benefit patients by delivering personalized medicine, for example by adjusting the dose of medication in accordance to the subject's placebo susceptibility or selecting a different class of drugs for the individualized patient.

Our study illuminates the persistent failure to identify reliable characteristics of placebo responders [50], [51], [52]. Beyond using a no-treatment control another unique feature of our study was the inclusion of an augmented therapeutic relationship. The therapeutic relationship is considered an active component of non-specific placebo treatment [30]; its presence may be needed to identify reliable predictors of the placebo effect [53]. Prior studies did not include an arm in which the placebo was administered within the context of an augmented therapeutic relationship. Indeed, many of these studies were done with volunteer subjects in experimental rather than clinical contexts in which there was no therapeutic relationship at all. Although this unique design of our study is an asset, it also poses a limitation on using other studies to retrospectively validate our findings, as most clinical trials do not include a no-treatment control. Another limitation of this study is its small sample size and the absence of a power calculation. Given that our study was a secondary data analysis of an existing dataset, the sample size was already fixed. Although a small sample size increases the risk of a type II error (failing to find a significant difference that exists in the population), it does not challenge the validity of results showing significant effects. Hence, our results deriving from a clear hypothesis are still reliable.

It is unlikely that a single locus like COMT, fully accounts for a complex behavioral phenotype like placebo response. However, given that COMT val158met is a functional polymorphism, which results in a three to four-fold reduction in prefrontal dopamine, it has a greater potential to contribute to behavioral variability than other non-functional or noncoding polymorphisms. That COMT has been implicated in multiple disorders i.e. major depressive disorder, Parkinson's and pain, might be deemed as a weakness in its candidacy for a placebo response marker. However we argue that this wide association with disease and drug treatments might be why it is such a powerful potential target for modulating placebo effects.

IBS is a functional condition characterized by a diversity of symptoms measured by subjective instruments, which also poses another limitation to our study. It should be noted though, that placebo treatment is especially thought to influence the experience and perception of subjective symptoms such as depressed mood and pain, rather than directly modifying disease pathophysiology [54], [55].

Finally, the power of the patient-doctor interaction to enhance healing is one of the hallmarks of clinical medicine, and increasingly resources are devoted to teaching clinicians how to communicate warmth and caring to their patients [56], [57]. Our findings reinforce this healing benefit for met/met patients and raise an interesting question. Despite their best efforts, many a warm and caring physician has had patients that seemed to derive minimum benefit from their empathic attentions. Our findings that val/val patients are less influenced by the placebo treatment, regardless of whether it is delivered in an augmented or limited context, potentially shed some light on this clinical challenge.
